# Lymphatic, blood vessel and perineural invasion identifies early-stage high-risk radically resected gastric cancer patients

**DOI:** 10.1038/sj.bjc.6603286

**Published:** 2006-08-01

**Authors:** M Scartozzi, E Galizia, L Verdecchia, R Berardi, F Graziano, V Catalano, P Giordani, D Mari, R R Silva, C Marmorale, C Zingaretti, S Cascinu

**Affiliations:** 1 The Departments of Clinica di Oncologia Medica, Azienda Ospedaliera Ospedali Riuniti, Università Politecnica delle Marche, Via Conca, 60020, Ancona, Italy; 2 Istituto di Medicina Clinica e Biotecnologie Applicate-Oncologia Medica, Università Politecnica delle Marche, Ancona, Italy; 3 Oncologia Medica, Ospedale di Urbino, Urbino, Italy; 4 Oncologia Medica, Azienda Ospedaliera S Salvatore, Pesaro, Italy; 5 Oncologia Medica, Ospedale di Fabriano, Fabriano, Italy; 6 Clinica di Chirurgia Azienda Ospedaliera Ospedali Riuniti, Università Politecnica delle Marche, Ancona, Italy; 7 Chirurgia, Azienda Ospedaliera S Salvatore, Pesaro, Italy

**Keywords:** gastric cancer, lymphatic and vascular invasion, prognostic factors

## Abstract

The availability of different treatment options for radically resectable gastric cancer reopened the question of treatment selection and correct definition of high-risk categories. Lymphatic, blood vessel and perineural invasion (LBVI/PNI) seem to possess the necessary potential to provide useful information for the clinical management of this disease. Seven hundred and thirty-four patients with advanced gastric cancer who underwent curative gastrectomy were analysed according to the presence of LBVI/PNI. Patients were divided into two groups: group A for patients with LBVI/PNI (189 patients 26%) and group B for patients without LBVI/PNI (545 patients, 74%). The disease-free survival (DFS) for patients in group A was 32.1 months, whereas it was not reached for patients in group B (*P*=0.0001); the median overall survival was 45.5 months for patients in group A, whereas it was not reached for patients in group B (*P*=0.0001). At multivariate analysis, the presence of LBVI/PNI appeared an independent prognostic factor for DFS and OS. Our results were confirmed in subgroup analysis, separately considering stage I and early gastric cancer patients with and without LBVI/PNI. Taken together, our findings suggest the importance of LBVI/PNI in gastric cancer as it may provide additional information for identifying patients at high risk, who may be candidates for further medical treatment after or before surgery.

Although many advances have been made in the diagnosis and treatment of gastric cancer, the global outcome for patients diagnosed with this disease is still disappointing with a 5-year survival rate not exceeding 30% of all cases in Western Countries (Bouvier *et al*, 2002; Nitti *et al*, 2003).

In operable gastric cancer, the extent of surgery and the value of adjuvant treatment remain matter of scientific debate, with surgery still representing the cornerstone of any curative procedure (Hohenberger and Gretschel, 2003; van de Velde and Peeters, 2003).

Although most clinical trials investigating adjuvant chemotherapy generated inconclusive results (Hermans *et al*, 1993; Earle and Maroun, 1999, Cascinu *et al*, 2005), it has been reported that a chemoradiotherapy adjuvant approach may improve the outcome of radically resected gastric cancer by lowering the incidence of local relapse (Macdonald *et al*, 2001).

More recently, the MAGIC trial by Cunningham *et al* (2005) strongly suggested that perioperative chemotherapy could represent an interesting treatment option for patients with resectable cancer of the stomach, but on the other hand only 43% of the patients were able to complete postoperative treatment. However, it is also important to point out that the value of any medical treatment administered either pre- or postoperatively should be considered according to the quality and extent of surgery, which has been shown to significantly influence the outcome of gastric cancer patients (Scartozzi *et al*, 2005, Smith *et al*, 2005).

The availability of various and different treatment options for radically resectable gastric cancer patients has consequently reopened the crucial question of both an accurate treatment selection and a correct definition of high-risk categories, which may help identifying subgroups of patients benefiting more from additional medical treatments after (or before) radical surgery.

Together with classic variables well known to have a definite prognostic value (i.e. T and N categories), many biological and pathological factors have been indicated as possible prognostic indicators, but often with conflicting results. Among the others, lymphatic and blood vessel invasion (BVI) seemed to possess the necessary potential to provide useful information for the clinical management of patients with gastric cancer.

In fact, it has been suggested that venous infiltration could be a valuable prognostic factor in gastric cancer involving muscolaris and subserosal layer (Fotia *et al*, 2004) and lymphatic and BVI was shown to be an independent risk factor for recurrence and poor prognosis in patients with node-negative cancer of the stomach (Hyung *et al*, 2002).

Moreover, in a prospective analysis among 459 patients lymphatic vessel invasion (LVI) was found to independently correlate with prognosis in patients with primary resected adenocarcinoma of the oesophagogastric junction (Burkhard *et al*, 2005).

Furthermore, some authors suggested that the presence of neural invasion could have a negative prognostic impact on prognosis of radically resected gastric cancer patients. (Tanaka *et al*, 1997; Duraker *et al*, 2003).

Nevertheless, data about lymphatic, blood vessel (LBVI) in early-stage and in lymph node-positive gastric cancer are lacking.

The objective of our study was to clarify the role of LBVI and perineural invasion (PNI) as prognostic factor in a group of patients who underwent surgical resection for gastric carcinoma, with the aim to serve as a tool for a more accurate and rational treatment selection.

## PATIENTS AND METHODS

### Patient selection

The patient's study population was selected from a central database, including 915 patients, affected by gastric cancer, operated in four different Institutions.

Classification of the T, N and M factors was made according to the numeric system introduced by the 5th TNM.

One hundred and eighty-one patients were excluded from our analysis because of incomplete clinicopathologic data or not radical surgery. As a result, 734 patients with advanced gastric cancer who had undergone curative gastrectomy were analysed according to the presence of LBVI/PNI.

We divided our patients into two groups: group A for patients with LBVI/PNI and group B for patients without LBVI/PNI.

Follow-up of both groups occurred at 3-month intervals for 2 years, then at 6-month intervals for 3 years and yearly thereafter. Follow-up consisted of physical examination, a complete blood count, chest radiography and ultrasound of the abdomen or computed tomography scanning as clinically indicated. The site and date of first relapse and the date of death were recorded.

### Data management and statistical analysis

Statistical analysis was performed with SAS software version 8.2. for Windows (SAS Institute Inc., Cary, NC, USA). The association between categorical variables was estimated by *χ*^2^ test. Survival distribution was estimated by the Kaplan–Meier method (Kaplan and Meier, 1958).

Significant differences in probability of relapsing between the strata were evaluated by log-rank test.

Cox's multiple regression analysis was used to assess the role of LBVI/PNI as prognostic factor adjusted for those variables resulted significant at univariate analysis.

Tested variables included sex (male *vs* female), age (<65 *vs* ⩾65 years), grade of tumour differentiation (well and moderately differentiated *vs* undifferentiated), depth of tumour infiltration (pT1–2 *vs* pT3–4, pT1 *vs* pT2–4, pT1–3 *vs* pT4), absence or presence of lymph node metastases (pN0 *vs* pN+), type of lymphadenectomy (extended *vs* limited, that is, >25 *vs* <25 removed lymph nodes), LVI (presence *vs* absence of lymphatic invasion), BVI (presence *vs* absence of BVI), PNI (presence *vs* absence of PNI) and LBVI/PNI (presence *vs* absence of LBVI/PNI).

Relative risk was defined as the ratio of the probability that an event (recurrence or death) would occur to the probability that it would not occur. The prognostic power of covariates was expressed by calculation of a relative risk with a 95% confidence interval (CI). A significant level of 0.05 was chosen to assess the statistical significance.

For statistical analysis, overall survival (OS) and disease-free survival (DFS) were defined, respectively, as the interval between surgery to death or last follow-up visit and as the interval between surgery to clinical progression or death or last follow-up visit if not progressed.

## RESULTS

Seven hundred and thirty-four patients were eligible for our analysis: 441 males and 293 females with a median age at diagnosis of 68 years (range: 30–94 years). Two hundred and thirty-seven patients had stage I, 152 stage II, 188 stage IIIA, 98 stage IIIB and 59 stage IV.

Among 734 patients with advanced gastric cancer who had undergone curative gastric resection, LBVI/PNI was present in 189 patients (group A, 26%), whereas it was absent in the remaining 545 patients (group B, 74%). Clinicopathological variables of both groups are summarised in Table 1.

**Figure 1:**
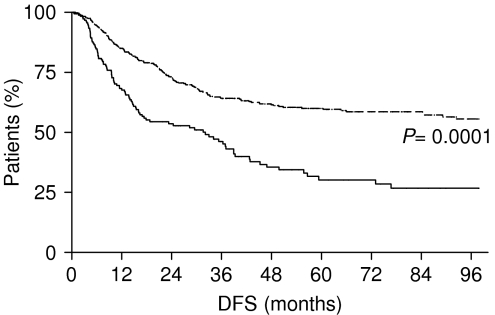
Disease-free survival of gastric cancer patients with LBVI/PNI (——— group A) and without LBVI/PNI (- - - - - - - - group B).

**Figure 2:**
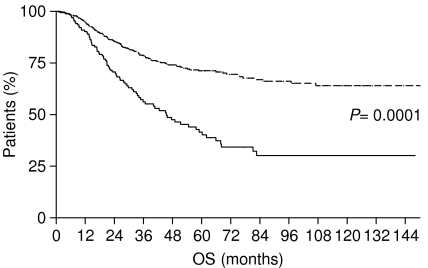
Overall survival of gastric cancer patients with LBVI/PNI (——— group A) and without LBVI/PNI (- - - - - - - - group B).

Only LVI was present in 73 patients (9.9%), only BVI was present in 50 patients (6.8%) and only PNI was present in 16 patients (2.1%). Lymphatic vessel invasion and BVI (LBVI) were present concurrently in 15 patients (2%), LVI and PNI were present concurrently in 18 patients (2.5%) and BVI and PNI were present concurrently in 17 patients (2.3%). At univariate analysis, pT stage, pN stage, number of resected lymph nodes, LVI, BVI, PNI and LBVI/PNI resulted prognostic factors for DFS and OS (Table 2). In particular, the DFS for patients in group A was 32.13 months, whereas it was not reached for patients in group B (*P*=0.0001) (Figure 1). The presence of LBVI/PNI also resulted determinant in OS with a median OS of 45.5 months for patients in group A, whereas it was not reached for patients in group B (*P*=0.0001) (Figure 2).

Groups A and B resulted statistically equivalent for all major clinicopathologic characteristics. However, we found a significant correlation between stage at diagnosis and presence or absence of LBVI/PNI. In fact, LBVI/PNI was more often found in gastric cancers in more advanced stages. Only 16.9% of gastric cancers with LBVI/PNI were diagnosed in stage I *vs* 37.6% of gastric cancers without LBVI/PNI (*P*=0.0001). Moreover, only 6.4% of gastric cancers without LBVI/PNI were diagnosed in stage IV *vs* 12.7% of gastric cancers with LBVI/PNI (*P*=0.0095).

The LBVI/PNI status was found related to the presence or absence of lymph nodes metastases: patients with LBVI/PNI more frequently showed the presence of lymph nodes metastases in comparison to patients without LBVI/PNI (*P*=0.0001).

At multivariate analysis, the presence of LBVI/PNI appeared an independent prognostic factor for DFS (hazards ratio (HR)=0.62, CI 0.48–0.80, *P*=0.0002), which was also influenced by extension within the gastric wall (HR=0.38, CI 0.29–0.51, *P*=0.0001), nodal involvement (HR=0.31, CI 0.22–0.43, *P*=0.0001) and by the type of lymphadenectomy (HR=0.60, CI 0.44–0.82, *P*=0.0012).

The presence of LBVI/PNI also resulted an independent prognostic factor affecting OS, which was also influenced by extension within the gastric wall, nodal involvement and by the type of lymphadenectomy (Table 3).

**Figure 3:**
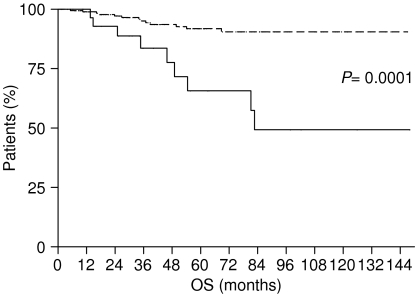
Overall survival of stage I gastric cancer with LBVI/PNI (———) and without LBVI/PNI (- - - - - - - -).

**Figure 4:**
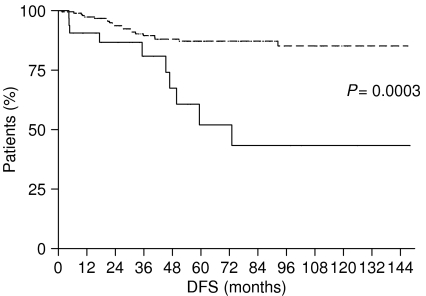
Disease-free survival of stage I gastric cancer patients with LBVI/PNI (———) and without LBVI/PNI (- - - - - - - ).

Our results were confirmed in subgroups analysis, separately considering stage I patients with and without LBVI/PNI. In fact, among stage I patients with LBVI/PNI, OS was 82.67 months, whereas it was not reached for those without invasion (*P*=0.0001) (Figure 3). Also, DFS appeared influenced by the presence or absence of LBVI/PNI in this latter group of patients as it was 73.03 months for patients with invasion, whereas it was not reached for patients without invasion (*P*=0.0003) (Figure 4).

**Figure 5:**
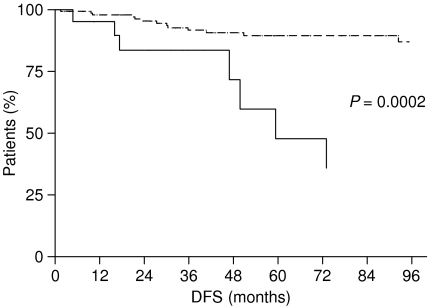
Disease-free survival of early gastric cancer patients with LBVI/PNI (———) and without LBVI/PNI (- - - - - - - -).

**Figure 6:**
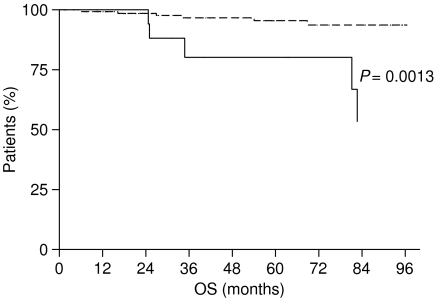
Overall survival of early gastric cancer patients with LBVI/PNI (———) and without LBVI/PNI (- - - - - - - -).

Moreover, when we considered patients affected by early gastric cancer (pT1 N0− or N+), we found similar results: patients with LBVI/PNI experienced a median DFS and OS significantly worse than patients without LBVI/PNI. Patients with LBVI/PNI had a DFS of 59.37 months, whereas it was not reached for patients without LBVI/PNI (*P*=0.0002) (Figure 5). Median OS was not reached neither for patients with LBVI/PNI nor for those without LBVI/PNI, but the difference was statistically significant (*P*=0.0013) (Figure 6).

**Figure 7:**
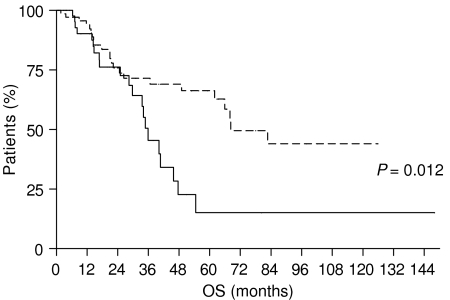
Overall survival of gastric cancer patients with BVI (———) *vs* gastric cancer patients with LVI (- - - - - - - -).

**Figure 8:**
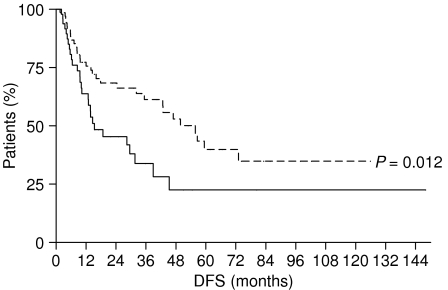
Disease-free survival of gastric cancer patients with BVI (———) *vs* gastric cancer patients with LVI (- - - - - - - -).

In order to evaluate the difference between LVI and BVI, as prognostic factors in gastric cancer patients, we compared OS and DFS of patients with LVI *vs* those of patients with BVI. We observed that patients with BVI had a worse outcome in comparison with patients with LVI: the OS of patients with LVI was 68.2 months, whereas that of patients with BVI was 35.87 months (*P*=0.012) (Figure 7); DFS was 55.77 months for patients with LVI and 15.37 months for those with BVI (*P*=0.012) (Figure 8).

## DISCUSSION

Although most clinical trials investigating adjuvant chemotherapy generated inconclusive results (Hermans *et al*, 1993; Earle and Maroun, 1999; Cascinu *et al*, 2005), the trial by Cunningham *et al* (2005) (the MAGIC trial) has re-opened the debate about chemotherapy for operable gastric cancer patients. In this study, 503 patients affected by adenocarcinoma of the stomach, oesophagogastric junction or lower oesophagus were randomised to perioperative chemotherapy or surgery alone. The authors of this study demonstrated that perioperative chemotherapy was able to significantly improve progression-free survival and OS. However, in the chemotherapy arm only 43% of patients completed the postoperative planned programme, probably as a consequence of the fact that gastric cancer patients have been often shown to be hardly compliant to postoperative chemotherapy (Cascinu *et al* 2005). On the other hand, it has also been previously reported that a chemoradiotherapy adjuvant approach may improve the outcome of radically resected gastric cancer by lowering the incidence of local relapse (Macdonald *et al*, 2001).

The availability of various and different treatment options has consequently reintroduced the crucial question of an accurate treatment selection and a correct definition of high-risk categories, which may help identifying subgroups of patients benefiting more from additional medical treatments after (or before) radical surgery.

Our findings suggest that the presence or absence of LBVI/PNI is an important aspect influencing the clinical outcome of gastric cancer patients, who underwent radical surgery and, more interestingly, it appeared an independent prognostic factor affecting DFS (*P*=0.0002) and OS (*P*=0.0001), which were also influenced by variables already known to represent important prognostic factors such as the extension within the gastric wall, nodal involvement and the type of lymphadenectomy (Scartozzi *et al*, 2005; Smith *et al*, 2005).

In the present analysis, we found a statistically significant difference in stage at diagnosis, DFS and OS between patients with LBVI/PNI and those without LBVI/PNI. In fact, the median DFS for patients in group A was 32.1 months, whereas it was not reached by patients in group B (*P*=0.0001); the median OS was 45.5 months for patients in group A, whereas it was not reached for patients in group B (*P*=0.0001). At multivariate analysis, the presence of LBVI/PNI appeared an independent prognostic factor for DFS and OS. Our results were confirmed in subgroups analysis, separately considering stage I and early gastric cancer patients with and without LBVI/PNI.

Our observations seem to integrate well to what has been already suggested by other studies hypothesising that LBVI/PNI may represent a prognostic factor in oesophageal squamous cell cancer and gastric cancer and that the prognostic value of these factors is not influenced by tumour stage, grade of differentiation or lymph node involvement (Gabbert *et al*, 1991; Kooby *et al*, 2003; Burkhard *et al*, 2005).

Our data in early gastric cancer patients are of particular relevance. Lymphatic, blood vessel and perineural invasion was, in fact, able to identify subgroups of patients with extremely different clinical outcome among cases usually considered at a low risk of recurrence, thus offering an effective tool for treatment selection and prognostic stratification in these cases.

Although prospective studies are needed, taken together our findings underline the importance of a careful search for LBVI/PNI in gastric cancer patients as it may provide additional useful information for identifying patients who are at high risk and who may be candidates for further medical treatment after or before surgery.

## Figures and Tables

**Table 1:** Patients characteristics

	**Whole group**	**Group A (%)**	**Group B (%)**
Number	734	189 (26)	545 (74)
Age (range)	68 (30–94)	69 (35–92)	67 (30–94)
			
*Sex*			
Male	441	105 (55.6)	336 (61.7)
Female	293	84 (44.4)	209 (38.3)
			
*Stage*			
I	237	32 (16.9)	205 (37.6)
II	152	40 (21.2)	112 (20.6)
IIIA	188	58 (30.7)	130 (23.8)
IIIB	98	35 (18.5)	63 (11.6)
IV	59	24 (12.7)	35 (6.4)
			
*pT stage*			
pT1	175	21 (11.1)	154 (28.3)
pT2	150	39 (20.6)	111 (20.4)
pT3	374	115 (60.9)	259 (47.5)
pT4	35	14 (7.4)	21 (3.8)
			
*pN stage*			
pN0	301	46 (24.3)	255 (46.8)
pN1	269	83 (43.9)	186 (34.1)
pN2	126	41 (21.7)	85 (15.6)
pN3	38	19 (10.1)	19 (3.5)
			
*Istopathology*			
Diffuse	90	30 (15.8)	60 (11.0)
Intestinal	290	81 (42.9)	209 (38.4)
Signet cells	56	9 (4.8)	47 (8.6)
Diffuse+signet cells	66	17 (9.0)	49 (9.0)
Other	232	52 (27.5)	180 (33.0)

**Table 2:** Significant prognostic factors for OS at univariate analysis

**Factor**	**OS (months)**	**Relative risk**	**95% CI**	***P*-value**
*pT stage*				
pT 1	NR	0.1339	0.2184–0.3970	0.0001
pT 2–4	68.2			
pT1–2	NR	0.2773	0.2177–0.3826	<0.0001
pT3–4	55.1			
pT1–3	NR	0.3201	0.06029–0.3019	<0.0001
pT4	34.5			
				
*pN stage*				
N0	158.5	0.2611	0.2259–0.3948	<0.0001
N+	65.3			
				
*Resected lymph nodes*				
<25	58.8	1.535	1.023–2.117	0.0371
>25	84.8			
				
*Lymphatic invasion*				
Yes	63.5	1.900	1.482–3.423	0.0001
No	148			
				
*Blood vessel invasion*				
Yes	35.9	3.179	3.914–11.60	<0.0001
No	142.1			
				
*Perineural invasion*				
Yes	33	3.178	3.830–13.62	<0.0001
No	150.8			
				
*LBVI/PNI*				
Yes	45.5	2.459	2.190–4.333	0.0001
No	NR			

CI, confidence interval; LBVI/PNI, lymphatic, blood vessel and perineural invasion; NR, not reached; OS, overall survival.

**Table 3:** Significant prognostic factors for OS at multivariate analysis

**Factor**	**Relative risk**	**95% CI**	***P*-value**
*pT Stage*			
pT 1	0.3178	0.1638–0.6165	0.0007
pT 2–4			
pT1–2	0.5550	0.3835–0.8032	0.0018
pT3–4			
pT1–3	0.5327	0.3218–0.8819	0.0143
pT4			
			
*pN Stage*			
N0	0.4638	0.3256–0.6607	<0.0001
N+			
*Resected lymph nodes*			

<25	0.6766	0.4686–0.9769	0.0371
>25			
			
*Lymphatic invasion*			
Yes	0.6846	0.4861–0.9641	0.0300
No			
			
*Blood vessel invasion*			
Yes	0.5216	0.3595–0.7567	0.0006
No			
			
*Perineural invasion*			
Yes	0.7093	0.4599–0.9393	0.0402
No			
			
*LBVI/PNI*			
Yes	0.52	0.39–0.69	0.0001
No			

CI, confidence interval; LBVI/PNI, lymphatic, blood vessel and perineural invasion; OS, overall survival.
